# Ion Lithography of Single Ions Irradiation for Spatially Regular Arrays of Pores in Membranes of Polyethylene Terephthalate

**DOI:** 10.3390/nano12223927

**Published:** 2022-11-08

**Authors:** Mariapompea Cutroneo, Vladimir Hnatowicz, Anna Mackova, Petr Malinsky, Romana Miksova, Giovanni Ceccio, Jan Maly, Jiří Smejkal, Marcel Štofik, Vladimir Havranek

**Affiliations:** 1 Nuclear Physics Institute AS CR, Hlavni 130, 25068 Rez, Czech Republic; 2 Department of Physics, Faculty of Science, University of J. E. Purkyně, Pasteurova 3544/1, 40096 Ústí nad Labem, Czech Republic; 3 Centre of Nanomaterials and Biotechnology, Faculty of Science, Jan Evangelista Purkyně University in Ústí nad Labem, 40096 Ústí nad Labem, Czech Republic

**Keywords:** polyethylene terephthalate, ion lithography, membrane, STIM, pore size-shape simulation, microfluidics, organ-on-chips

## Abstract

Routinely, in membrane technology, the decay from radioactive particles or the bombardment of ions with MeV energy per nucleon have been employed for the production of narrow and long pores in membranes. Presently, the ion lithography is proposed to make the fabrication cost more affordable. It is prospective for the use of medium capacity accelerators making more feasible the fabrication of customized membranes. Thin polyethylene terephthalate foils have been patterned using 12 MeV O^5+^ ions and then processed to obtain good aspect ratio ion track pores in membranes. Pores of micrometric diameter with the following profiles were fabricated in the membranes: truncated cone, double conical, ideal cone, and cylindrical. Monitoring of the shape and size of pores has been attempted with a combination of Scanning Transmission Ion Microscope and a newly designed simulation program. This study is focused on the use of low-energy ions, accomplished in all laboratories, for the fabrication of membranes where the pores are not randomly traced and exhibit higher surface density and negligible overlapping than in membranes commonly manufactured. The good reproducibility and the ordered pore locations can be potentially utilized in applications such as microfluidics and organ-on-chip microsystems, where cells growing over porous substrates are used in simulation of biological barriers and transport processes.

## 1. Introduction

The development of membranes for medical separation processes dates back to 1945 when W.J. Kolf [1] demonstrated the first successful artificial kidney in The Netherlands. 

In this case, 20 years ago, more than 800,000 people were counted as being assisted by artificial kidneys and more than one million people deal with open-heart surgery each year, based on the fabrication of the membrane blood oxygenator [2].

In 1964, after the recognition of particle track-etching in thin foil material [3], the use of nuclear tracks made the fabrication of membranes competitive in the track membrane industry allowing for the expanded use of the membranes in cell culture activities and in filtration [4,5]. In recent years the interest in the design of porous material to improve and modulate cell growth [6] emerged to promote the construction of functionalized scaffolds for tissue regeneration [7] and to design a microfluidic/organ-on-chip devices for cell cultivations, migration assays and organ simulations [8,9].

Although today membranes are commonly fabricated by irradiation of polymeric foil with fragments from the fission of heavy nuclei or with ions at energies of a few MeVs per nucleon [10] both procedures count inconveniences. In addition to the high production cost, the production in nuclear reactors can be responsible for contamination of the membranes with radioactive products, while the stability of the particles flux from an accelerator for the use of very high ion energy, of the order of hundreds of MeV, is lower than that in a reactor [11]. Further requirements for the fabrication of membranes using high energy ions are low ion fluence and large scanning area which can be accomplished only in big facilities such as in Brookheaven, Dubna, Louvain-la-Neuve and Berlin. Although the handling of the diameter, shape, orientation and number density of the pores in the membranes can be claimed using heavy ions kinetic energy [12], none of the mentioned approaches are feasible for the construction of membranes with regular pores located in selected positions. 

From this perspective, the use of ion lithography could be beneficial to obtain customized patterns with smooth side wall channels without the use of masks or sacrificial layers [13], to promote the use of common accelerators generating ions with energy up to tens of MeVs, and to fabricate reproducible membranes with well separated pores in the micrometric scale and density of about 10^6^ ions/cm^2^. The only drawback in the use of ion lithography is the size of the produced pores which is far from the best values of 15–20 nm in diameter mentioned using other methods [14] but still well-suited for biological applications where pore sizes ranging between a few microns to hundreds of microns are required.

The transfer of energy from heavy ions when passing through a thin foil of polymer occurs mainly through excitation and ionization, inducing highly reactive transient products in particular: free radicals, molecular ions, hot atoms, and unsaturated molecular fragments [15]. Afterwards, the transients recombine or react with surrounding matter and finally, the so-called latent track, a cylindrical region along the ion trajectories with altered structure, composition and physico-chemical properties, is formed. The concentration of the transients and then the final degradation products is considered an increasing function of the energy locally deposited by the ion and the diameter of the central track region, proportional to the ion instantaneous velocity [15].

However, the slowing down of energetic ions moving in the target is due to both the electronic (Se) and the nuclear (Sn) stopping powers. The latter is generated from collisions between the ion and nuclei of the target atoms partially screened by the core electrons [16]. Conventionally, the Se dominates at higher ion energies while the nuclear stopping is dominant only for ions of relatively low energy (Ekin < 100 keV/nucleon). The structural modifications due to electronic stopping are normally more significant close to the surface, whereas defects due to nuclear stopping appear inside the material (several micrometers) at the ends of ion projected ranges. 

In this study, 12 MeV O^5+^ ions were used to irradiate a thin foil of polyethylene terephthalate. In particular, the ion current has been significantly decreased to improve the focusing of the ion beam to drive high ion energy loss in the track when bombarded at a precise point in the foil depending on the selected pattern. The aim of the proposed procedure is to reduce the risk of overlapping the ion tracks and obtain micrometric pores in regular positions which in combination with membrane thickness can affect the cell behavior (attachment, alignment, growth) [17]. Given the criticality of the pore shape, which is linked to the transport properties of ions, molecules, cells, liquids, gases, particles, solutes, and electrolytes, an accurate characterization of the narrow pores is demanding and still very difficult. The common approaches to evaluate the pore geometry are based on the use of Scanning Electron Microscopy (SEM) [18] or observation of metal replicas through Field Emission Scanning Electron Microscopy (FESEM) [19,20]. Typically, SEM provides the measure of the geometry of the individual pore using computer image analysis. Here, the complex contrast of the SEM image and the high depth of focus make it quite impossible to distinguish between structures at different heights. In the case of FESEM when high-quality replicas are used, only limited information can be provided because the images cannot display the details of the pore, whose radius can be several nanometers small. 

An alternative approach to determine the pore size and geometry, particularly in PET where the ion tracks exhibit complex damage structure [21] could be the combination of the Scanning Transmission ion Microscopy [22] and the dedicated simulation program designed and improved in our laboratory and tested for the first time in this study.

## 2. Materials and Methods

**Figure 1:**
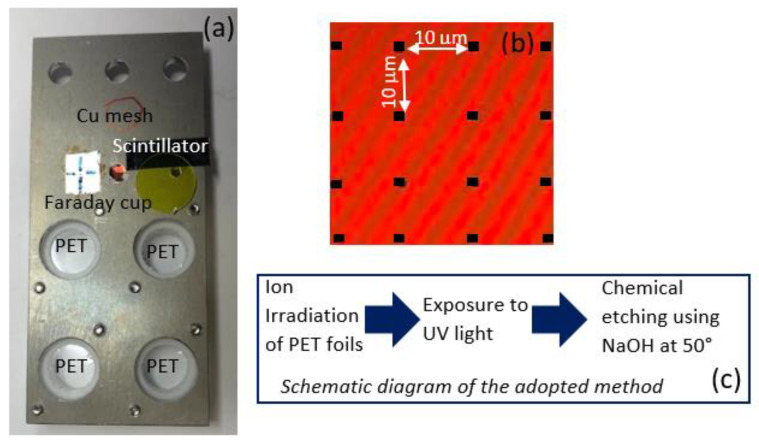
Sketch of the holder used for the ion irradiation of PET (**a**), the drawing of the pattern which has been reproduced on the PET membranes (**b**) and the schematic diagram of the membrane fabrication (**c**).

The PET foils with a size of 15 mm × 15 mm were irradiated with 12 MeV O^5+^ ions at the Tandetron accelerator [23]. The average energy loss (dE/dx) of the employed ions and kinetic energy in the 6 µm PET foil considering its composition (H = 36%, C = 45% and O = 18%) and its density of 1.397 g/cm^3^, is 1452.0 keV/µm for the electronic stopping power and 1.8 keV/µm for the nuclear stopping power in agreement with SRIM program [24]. Figure 1a shows the holder where the PET foils have been located together with the Cu mesh and the scintillator used for the evaluation of the resolution and for the focusing of the beam, respectively.

### 2.1. Ion Irradiation

The PET foils have been placed in a customized holder (see Figure 1a) inside the vacuum chamber, perpendicular to the ion beam axis on a motorized *x*-*y*-*z* stage. The divergence of the beam distribution over the target was less than ±10%. Energetic O^5+^ ions of about 12.0 MeV have been focused to a spot size of about 1 µm with a resolution of 0.6 µm. The main rate of incoming ions has been detected prior and after the irradiation by Si PIN diode detector (S14606-Hamamatsu) in the absence of the foil. Samples have been irradiated with a beam aperture adjusted to 5 µm × 10 µm in the horizontal and vertical directions and following two different configurations, those being random ions in the foil and random ions following a pattern.

(1) In the first case, the mean of incoming ions passing through the beam aperture has been set at 50 ions/s and the irradiation time at 200 s/pattern. The random ions delivered a total ion fluency of 1 × 10^4^ ions/mm^2^ scanning over a PET surface of 1000 µm × 1000 µm.
(2) In the second case, the incoming ions have been released following the pattern illustrated in Figure 1b with the following conditions: 200 s/pattern (i.e., 20 ms/pixel) which is equal to an arrival in the pattern of 10000 ions; 100 s/pattern (i.e., 10 ms/pixel) which is equal to an arrival in the pattern of 5000 ions.

The ion beam has been guided vertically over a grid pattern composed of 99 × 99 dots dispersed at 10 μm intervals (see Figure 1b); the ions traverse the pattern from top to bottom and back again over an area of 1000 μm × 1000 µm. The patterns with 10000 and 5000 ions have been irradiated taking into account the random time distribution of incoming ions. This means that the probability of hitting single dots in the pattern is governed by the Poisson distribution where *λ* is the expected number of successes given by:

$$\lambda =\frac{total\;number\;of\;incoming\;ions}{number\;of\;dots}$$

The Poisson distribution indicates the discrete probability of a given number of events occurring in a fixed interval of time or space under the condition that the events occur with a known constant average rate and independently of the time since the last event.

Accordingly, assuming the Poisson model is appropriate, in the condition of 10000 ions, there is about a 37% probability of no arrival of ions, about 37% of an additional arrival of a single ion and approx. In this case, there is a 18% probability for the arrival of two additional ions in a 20 ms/dot. In the condition of 5000 ions, there is about a 60% probability of no arrival of ions, about 30% for an additional arrival and approx. Additionally, there is a 7% probability for the arrival of two additional ions [25] in a 20 ms/dot.

The *x*-*y* beam scanning, the released dose, depending on the scan velocity and the number of loops, and the blanking, denoting the time necessary during the skipping of the beam from one point in the pattern to the next, has been controlled by Scan5.vi software written in LabView code [26].

### 2.2. UV Irradiation

Following the ion irradiation, both sides of the 6 µm thick PET have been subjected to UV exposition treatment for 5 h before the chemical etching using a Herolab GmbH Laborgerate (Wiesloch, Germany) UV lamp working at an intensity of 4 W/m^2^ in the range 254–361 nm. The samples were immersed in 5N (NaOH) solution at 50 ± 0.5 °C for 2.5 h.

The aim of the exposition to UV is to induce the photo oxidation [27] and increase the track etch ratio because the presence of oxygen at the room atmosphere promotes the sensitivity of ion-irradiated material to the etching along the ion tracks through the oxidation of the radicals generated during the irradiation.

### 2.3. Scanning Transmission Ion Microscopy

The micro ion beam has been additionally used as a probe for the evaluation of the pores’ breakthrough and size in the fabricated membranes [28]. The cross-sectional view of the 2D pores has been obtained using the well-established non-destructive method of Scanning Transmission Ion Microscopy (STIM). The lateral energy loss of ions crossing the sample has been converted into the local thickness of the sample taking into account the known energy loss for unit thickness of the used material. A scanning 3.0 MeV He^2+^ beam, focused to a size better than 0.5 µm, was used for analysis as explained in the literature [29]. The beam current was set to about 1–2 K particles/s. The helium beam penetrates the PET foil with an energy loss but without creating damage along its trajectory. This occurs thanks to its very low fluence (~10^6^ per pattern) and high energy (3.0 MeV). The 2D maps of the lateral thickness distribution have been acquired in scanning windows of 500 μm × 500 μm for a better view of the general pattern and of 75 µm × 75 µm for a detailed view of the pores.

### 2.4. Simulation Program for Pore-Shape Evaluation

The TrackHH code is the upgraded version of the previous TOM [30,31] used for the characterization of the radial structure of latent tracks from the energy spectrum of the alpha particles produced by 241Am α-source, with 5.486 MeV main alpha energy line transmitted from a membrane. The spectra evaluation was challenged by several alpha particle groups of different energies emitted by 241Am (that is 855 of 5486 keV, 135 of 5443 keV and 2% of 5388 keV). The new TrackHH tested in the present study uses Monte Carlo simulation to compute the energy spectra output from the Si PIN diode detector (S14605- Hamamatsu) located behind the PET membrane irradiated by monoenergetic ions. Since the particle energy loss is influenced by several parameters such as the ion beam divergence, incidence angle of the beam, thickness, density and structure of the PET foil, number of tracks in the foils and pore geometry, the transmission spectrum includes information about the foil inhomogeneities. The estimation of the pore size and shape is obtained through the comparison of the experimental energy spectrum and the simulated one computed using the aforementioned parameters as free parameters in Monte Carlo simulation.

### 2.5. In Vitro Cell Culture

Immortalized Human Brain Microvascular Endothelial Cells (IM-HBMEC, Innoprot, Bizkaia, Spain) were used as the model cell line for the experiment. The cell line was grown in the Endothelial Cell Medium (ECM) (P60104, Innoprot). The cells were maintained in the culture flasks (surface area 25 cm^2^, maximum volume 10 ml) coated with fibronectin 4 µg/cm^2^ and cultured in the cell culture incubator (CelCulture CO_2_ incubator, Esco Micro Pte. Ltd., Singapore) at 37 °C in a humidified atmosphere (95%) including 5% CO_2_.

The cells were harvested and used in experiments two times per week after obtaining 40–60% confluence. Before passaging the cells, the medium was removed, and the cells were rinsed with 5 ml of PBS buffer (Phosphate Buffered Saline, P4417, Sigma-Aldrich spol. s.r.o, Prague, Czech Republic). The cells were trypsinized with 0.5 ml of trypsin solution (Trypsin-EDTA, T4174, Sigma-Aldrich spol. s.r.o, diluted 10×) for 5 min. The cells were then suspended in 2 mL of the cell culture medium and loosened apart by repeated pipetting. The cells left in the flask were mixed with 5 ml of the new media and left for incubation. The number of viable cells was determined by trypan blue exclusion on a CellDrop FL (DeNovix Inc., Wilmington, DE, USA).

The cell seeding was performed using 100µL of cell suspension pipetted onto the membranes at concentration 200,000 cells/mL. Membranes were then submerged in 1 mL of ECM medium and incubated in a cell incubator for 48 h.

### 2.6. Cell Imaging and Data Evaluation

The cells were stained using a staining solution consisting of 1 µM CellTracker Orange CMRA Dye (Invitrogen™) and of Hoechst 33342 2 µg/mL. The cells were stained for 45 min in the incubator. The cells on membranes were then washed 3 times with PBS and submerged back into the fresh ECM medium.

The clls on the surface of the membranes were documented using an inverted fluorescence microscope Olympus IX71 (Olympus Czech Group, s.r.o., Czech Republic) coupled with the CCD camera QImaging retiga 2000R (QImaging, Surrey, BC, Canada) and the excitation source pE-4000 (CoolLED Ltd., Andover, UK). The cells were imaged with the objective Olympus UPLFLN 4×. For Hoechst labeled cells a 360–370 nm excitation filter, 400 nm dichroic mirror, and 420–460 nm emission filter (Chroma Technology Corp, Rockingham, VT, USA) were used. For CMRA stained cells, a 530–550 nm excitation filter, 570 nm dichroic mirror, and 580–630 nm emission filter (Chroma Technology Corp, Rockingham, VT, USA) were used.

Count and Measure module of CellSens Dimensions 1.11 software, built 111222201 (Olympus Czech Group, Inc., Prague, Czech Republic) was used for the automated cell counting. ROI that covered the square of holes pattern was created. The minimum of the manual threshold on ROI was set at a value of 40, the minimum pixel count to register an object was set to 12, and the watershed function was turned on. After automated cell counting, every picture was inspected by the operator to ensure that automated cell counting finished without any errors.

### 2.7. Preparation of Samples for SEM Microscopy

The membranes with cells were submerged in 2.5% glutaraldehyde (Merck KGaA, Darmstadt, Germany) in 0.1 M Sodium Cacodylate pH 7.2 (Merck KGaA, Darmstadt, Germany) for 1 h at 4 °C. The membranes were washed 3 times with 0.1 M phosphate buffer. Alcohol dehydration was performed by stepping up the ethanol % diluted in distilled water as follows: 30% 50%, 70%, 80%, 90%, 95%, and 100% for 10 min each. The final 100% ethanol was replaced 3 times. The membranes were Critical Point dried using Leica EM CPD300 Automated Critical Point drier (Leica Microsystems, Wien, Austria) with settings: stirring 50%, CO_2_ in speed slow, CO_2_ in delay 2 min, Exchange speed 1, Exchange cycles 18, gas out heat slow, gas out speed slow 100%. Samples were then sputter coated with 20 nm gold (Q150T ES, Quorum Technologies, Lewes, UK) and imaged by scanning electron microscopy (SU5000 FE-SEM, Hitachi High-Tech Europe GmbH, Krefeld, Germany). The selected specimens were mounted on specimen stubs by double-sided adhesive carbon tape, placed into an electron microscope holder, and observed using an SE(L) detector in a high vacuum mode at various voltages and various magnifications (noted in each figure).

## 3. Results

**Figure 2:**
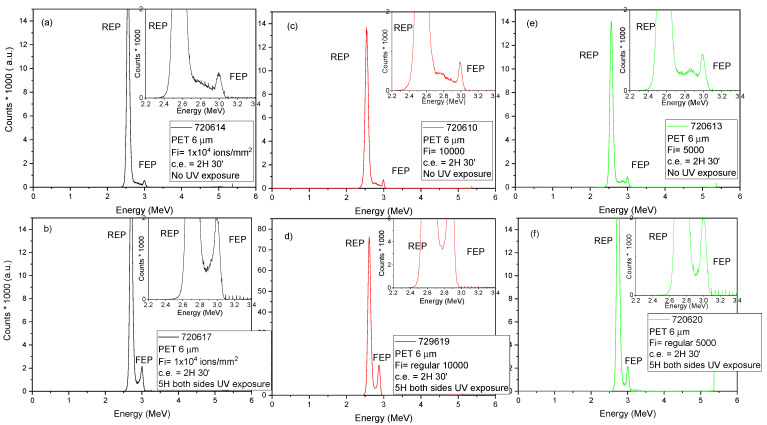
Spectra of the energy loss of He^2+^ ions at 3.0 MeV through 6 μm PET; random ions in foil without UV (**a**) and with UV (**b**), 10000 random ions in pattern without UV (**c**) and with UV (**d**) 5000 random ions in pattern without UV (**e**) and with UV (**f**).

The energy spectrum of transmitted He^2+^ ions at 3.0 MeV energy passing through the membranes has been provided by the Si PIN diode detector. Figure 2 shows the typical experimental energy spectra for membranes produced in PET 6 μm by random ions in the foil (see Figure 2a,b), 10000 random ions in pattern (see Figure 2c,d) and 5000 random ions in pattern (see Figure 2e,f).

In the transmitted experimental energy spectra two peaks are revealed, those being the Full Energy Peak (FEP) originating from particles transmitted without loss of energy through open channels (etched pores), and the Reduced Energy Peak (REP) from the particles transmitted through the whole thickness of the foil. The area between the FEP and the REP is related to particles with partial energy loss, and it reflects the changes in the pore size and shape at different depths. Their ratios evidence the unirradiated area over the porous area in the foil. The broad energy peak of the REP is ascribable to the particle straggling and multiple scattering.

The energy spectra of the 6 μm PET foils unexposed (see Figure 2a,c,e) to UV before the chemical etching, evidence irregular shapes in the area between the FEP and the REP.

That broad energy distribution corresponds to particles with partial energy loss, assignable to the energy loss of ions transmitted through both undamaged and free pore areas. Since it is affected by the shape and density of the pores it has been investigated for the evaluation of the pore shape [32]. 

### 3.1. TrackHH Simulation Processing

**Figure 3:**
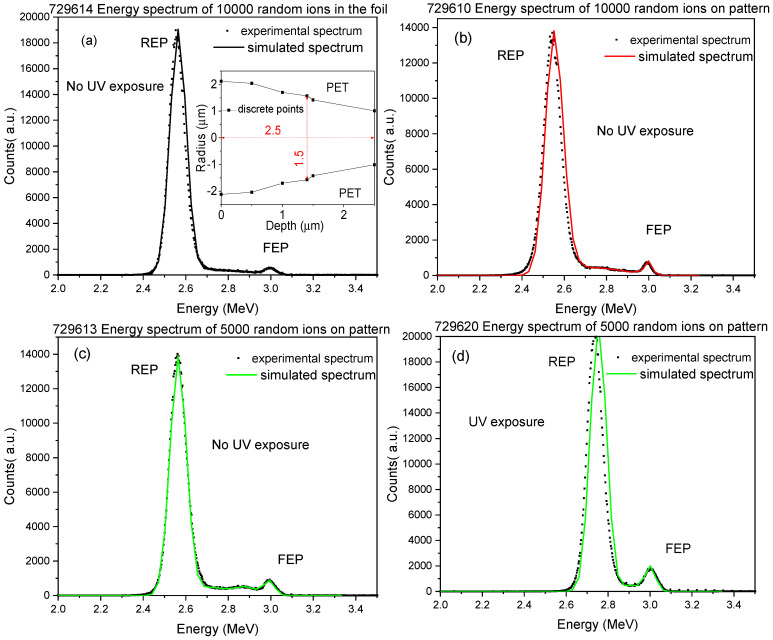
Experimental and simulated spectra obtained by TrackHH program for the membrane fabricated by ion irradiation without UV exposure (**a**–**c**) and with UV exposure (**d**). In the inset is reported the supposed pore shape related to the 1000 random ions in the foil.

Computer simulations have become a useful tool for the mathematical modeling of many natural systems not only in Physics. Our TrackHH code utilizes Monte Carlo simulation for the evaluation of the experimental spectra and the generation of simulated energy spectra of the transmitted ions for different pore shapes. The experimental energy spectra displayed in Figure 2 have been recognized and linked to proper files for ion energy loss, energy calibration of incident ions, density of the employed foil by TrackHH code. The TrackHH code has simulated the energy spectra of monoenergetic ions with randomly chosen trajectories passing through the pores of different shapes (conical, double conical, hourglass, cylindrical) and different densities (fully filled, partially etched, fully empty) through the point-by-point evaluation of the energy loss of transmitted ions and subsequent conversion into local thickness. The local pore radius is given as the function of the distance between the upper surface foil at several discrete points (see Figure 3a inset) and calculated by linear interpolation. The ion trajectory is assumed to be a straight line and the multiple scattering is negligible. During the processing, the particle trajectory is progressively incremented by a small constant step and tested at different pore positions while the ion energy loss is modified in agreement with SRIM. The track density given by the ratio between the REP and the FEP, the incident angle of the probing beam, and the pore shape file indicating the pore size at different depths, have been modified by the operator throughout the processing to obtain the overlapping of the experimental energy spectrum and the simulated one. Finally, the experimental spectra and the simulated ones have been compared to reconstruct the pore geometry, size and foil thickness. The good agreement between the experimental energy spectrum and the Monte Carlo simulation curve displayed in Figure 3 has been obtained using the following input parameters: 3.0 MeV energy of He^2+^ ions transmitted through a PET foil, ion beam incidence angle of 0°, a density of the tracks of about 10^4^ track/cm^2^, circular shape of pores with variable mean radius, the divergence of the ion beam of about 0.14°.

**Figure 4:**
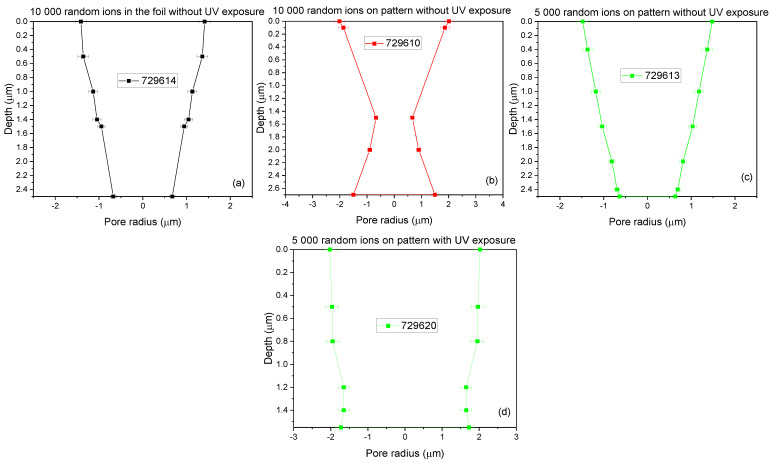
Model of the pores’ shape for 10000 random ions in foil (**a**), 10000 random ions on pattern (**b**), 5000 random ions in pattern (**c**) without UV exposure and 5000 random ions on pattern with UV exposure (**d**).

The ratio between REP and FEP reflects the values of the foil area and the pores one. The resulting shape displayed in the fabricated membrane obtained from the study of the area between FEP and REP carried out by TrackHH is depicted in Figure 4. The membrane created by irradiation of 10000 random ions in foil exhibits a truncated cone shape with a maximum diameter of 2.8 μm at the top surface, 1.4 μm at the bottom and depth of 2.5 μm (see Figure 4a). The membrane produced by irradiation of 10000 random ions on pattern shows a double conical shape with a base diameter of 3.8 μm at the top surface, 3.0 μm at the bottom and depth of 2.7 μm (see Figure 4b). 

Typically, the conical pore shape is expected when no UV exposure is used because it is responsible for an asymmetric etching setup [33].

The membrane obtained using 5000 random ions in pattern resembles an ideal cone shape with maximum and minimum diameters of 2.8 μm and 1.2 μm, respectively, and height of 2.5 μm (see Figure 4c). This is quite the opposite for all the patterns exposed to UV revealing regular cylindrical pore shapes, with maximum and minimum diameter of 4.2 μm and 3.6 μm, respectively, and height of 1.5 μm (see Figure 4d). The results suggest that the UV exposure induces decreasing of the hydrophobic binding influence of the surfactant adsorption, resulting in a more responsive chemical attack in the ion irradiated areas in agreement with the literature [34]. The error in the evaluation of the pore size is less than 10%.

### 3.2. Scanning Transmission Ion Microscope Analysis

Further estimation of the pore shape and size has been performed by a non-destructive analysis involving the use of 3.0 MeV, He^2+^ ions to convert the energy loss evaluation in thickness. The electronic energy loss for the 3.0 MeV helium ions passing through a PET foil is about 165.2 keV/μm and the nuclear energy loss is 12.89 keV/μm in agreement with SRIM [24].

**Figure 5:**
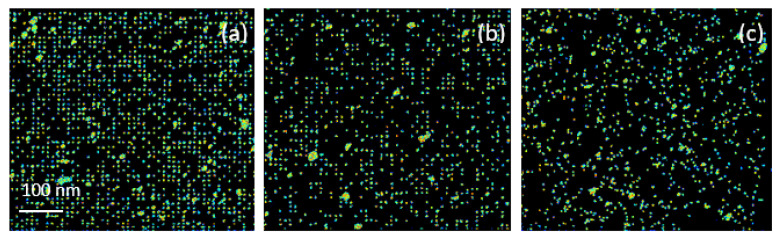
Stim maps obtained from 10000 random ions in a pattern (**a**), 5000 random ions in a pattern (**b**) and 10000 random ions on foil (**c**) all exposed to UV.

In the first slice (about 0.28 μm thick) the energy loss is nearly 50 keV and the penetration depth of He^2+^ ions in the PET is indicated to be about 14.0 μm. Several areas of the samples have been investigated to compensate for statistical fluctuation as a consequence of the straggling effects and to obtain a better estimation of the shape heterogeneity of pores. Figure 5 shows the 500 μm × 500 μm scan size of the fabricated membranes to clearly visualize the whole patterns. The more dense and regular pores of the 10000 random ions dispersed in a regular pattern are displayed in Figure 5a; the still acceptable regularity obtained using 5000 ions with the same pattern in Figure 5b and the total randomness of the pores, giving a perception of fewer, as produced by the 10000 random ions used on the foil in Figure 5c.

**Figure 6:**
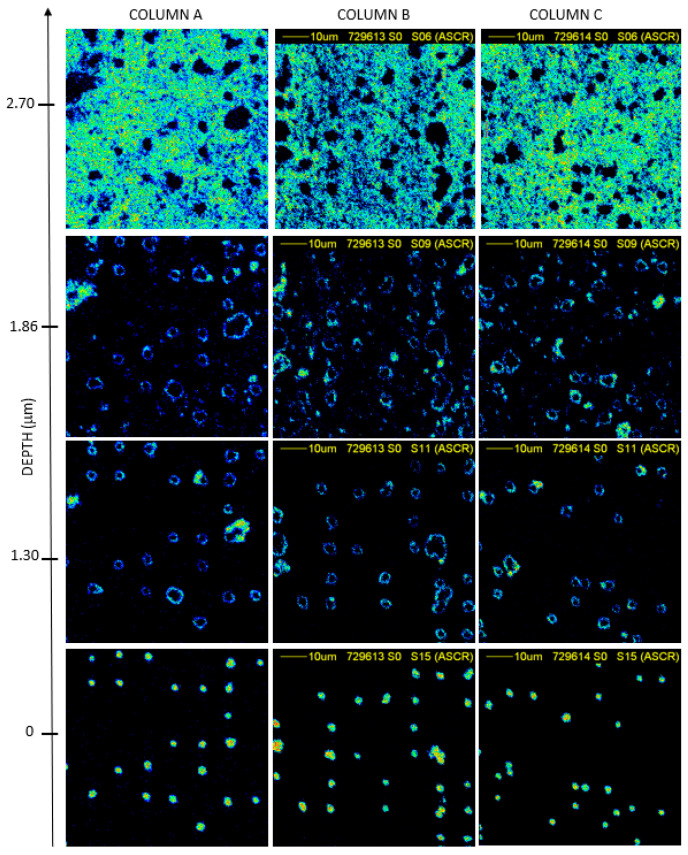
STIM images, 75 μm × 75 μm scan size, of the PET membranes obtained by irradiating with O^5+^; 5000 random ions in a pattern column **A** 10000 ions in a pattern column **B** and 10000 random ions in foil column **C** unexposed to UV.

**Figure 7:**
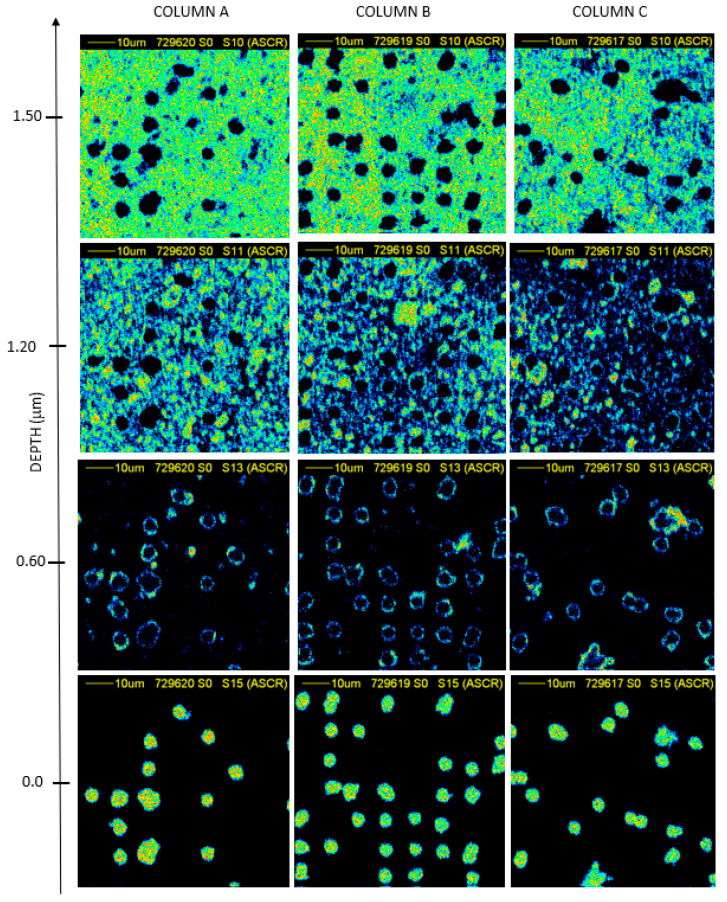
STIM images, 75 μm × 75 μm scan size, of the PET membranes obtained by irradiating with O^5+^; 5000 random ions in a pattern column **A**, 10000 random ions in a pattern column **B** and 10000 random ions in foil column **C** exposed for 5 h on both sides to UV.

The details of the contour of the pores and the surface morphology of the PET foils at 75 μm × 75 μm scan size is reported in Figure 6 and Figure 7.

The density maps of the transversal plane of the foils as a function of the depth, from the surface to the full thickness of the PET foil of 10000 random ions in foil (column A), 10000 random ions in pattern (column B), and 5000 random ions in pattern (column C) when the foils have been not exposed to UV are reported in Figure 6) and the foils have exposed to UV in Figure 7).

The thickness of the slices reported in the maps has been calculated from the lateral energy loss of ions crossing the foil and then converted into the local thickness knowing the energy loss for each slice of the used material. In Figure 6, the maximum thickness of the membranes is of about 2.70 μm, while in Figure 7 it is of about 1.5 µm indicating a reduction of 0.55% and 0.75% with respect the employed virgin PET, respectively. Both of the obtained values are in good agreement with the values obtained using TrackHH simulations indicating for the same set of membrane values of about 2.5 μm and 1.7 μm, respectively.

In addition to the final thickness of the membranes, the size of the pores produced on PET foils under ion irradiation seems to be strongly related to UV exposition as evidenced by the comparison between Figure 6 and Figure 7. The UV exposure induced a better etching ratio rate on the exposed membranes, while on the non-exposed different pore shapes have been displayed in agreement with the TrackHH simulations.

### 3.3. Imaging of Growing Cells on Membranes 

**Figure 8:**
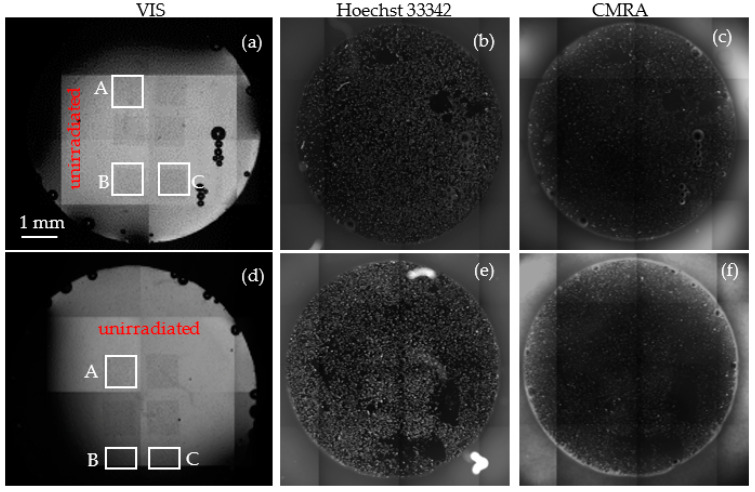
Optical images 4× magnification of the PET membranes obtained by irradiating with O^5+^ 10^4^ random ions in foil (see square A), 5000 random ions in a pattern (see square B), 10000 random ions in a pattern (see square C) in unexposed membrane (**a**–**c**) and in the UV exposed one (**d**–**f**).

The observation of cell growing on the surface of the membranes has been performed in the UV, VIS and NIR ranges (see Figure 8). The identification of cells in the UV and NIR has been promoted through their staining with cellTracker Orange CMRA Dye and Hoechst. The structures of interest showed in Figure 8 have been marked in white squares and labelled as A, B, C. Figure 8 displays 4× magnified optical images of the PET membranes obtained by irradiating with O^5+^ 10^4^ random ions in foil (see square A), 5000 random ions in a pattern (see square B), 10000 random ions in a pattern (see square C)) in unexposed membrane (see Figure 8a–c) and in the UV exposed one (see Figure 8d–f). 

Dense coverage of the growing cells regardless of type of PET treatment (exposed to UV (see Figure 8d–f) and membrane unexposed to UV (see Figure 8a–c)) was observed. Cells were able to grow on PET surface without modification with adhesion proteins, showing no significant differences between porous and pristine PET samples.

### 3.4. SEM Imaging of Growing Cells on Membranes

**Figure 9:**
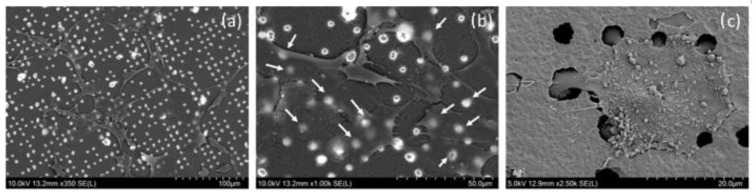
SEM images of cells growing on the porous PET membrane. (**a**) general overview on periodic array with growing cells; (**b**) pores under the cells in greater detail, arrows pointing to the pores under the cells; (**c**) the individual cell on the back side of the membrane. The cell has the protrusions of its cell membrane penetrating through 5 different pores to the other side of the membrane, which it migrated from.

In order to analyze the cell-material interaction in more detail, we acquired the SEM images of cells growing on pore arrays from both sides of membrane (see Figure 9). The size of the pores is an important parameter influencing the ability of cells to migrate through the pores. If the pores are small, the cells are not able to migrate to the other side of the membrane. If the pores are large enough, cells can migrate through the pores and grow on the other side of the membrane. Both of these phenomena can be actively used in different applications, as are, e.g., construction of microfluidic biological barrier models (e.g., blood-brain barriers) or where cell motility is monitored, for example in the migration assays of aggressive tumor cells. Therefore, precise control of pore size and their distribution is critical for such devices. Figure 9a, b shows that most of the cells were not able to penetrate through the ordinary pores and were able to overgrow them. In some cases, the individual cells were able to migrate through the larger defects in the membrane to the other side of membrane (see Figure 9c). 

## 4. Discussion

The demands for the development of customized membranes using common accelerators which are easily accessible and have lower running costs than the ones typically used led to the present study reporting on the fabrication of membranes consisting of micrometric size pores, realized using 12 MeV O^5+^ ions. One direct benefit derived from the use of the ion lithography technique has been the good control of the ion fluence and the high blanking time assisted in the fabrication of two different membranes containing random pores in the foil and regular pores in a selected pattern. In both cases, the merging of the neighboring tracks was minimized, and the size and shape of the pores were customized.

The proposed TrackHH simulation code provided a mean estimation of the pore size and shape depending on several parameters which cannot be precisely controlled. Since the critical parameters impacting the functionalization of the membranes are the pore size and shape, STIM measurements in conjunction with the TrackHH simulation program were performed. Both use the energy loss of transmitted monoenergetic ions passing through the membrane. The changes of the ion energy loss in the spectra reflected the alteration of the density in the ion tracks corresponding to more or less breached pores and in the variation of the foil thickness from 6 μm up to 1.5 μm.

The pores in all the membranes unexposed to UV were broken through, showing sizes ranging between 2.8 μm and 3.8 μm and shapes suggesting possible customization from truncate cone to double conical to ideal cone due to the asymmetric etching rate. On the contrary, the photo-oxidation induced on the membranes exposed to UV was responsible for the cylindrical shape in all membranes realized in all three different configurations and with a stable size of about 4.2 μm. The combined use of STIM and TrackHH showed a good conformity in the pore size and final foil thickness.

Moreover, their use, despite other microscopy techniques such as SEM, has proved advantageous. The high depth of focus and the contrast dominated by local sample orientation are not limitations because the distinction between polymer and pore is clear as well as the information depth defined by the thickness of the slice. Further advantages disclosed by the use are their negligible modification of the membranes in the reconstruction of the pore shape and easy applicability even when pores are not fully developed. Typically, track-etched membranes used for biological applications have a random distribution of pores with low surface density and overlapped pores. The in vitro cell culturing suggests that the physicochemical properties of the PET have been not negatively modified during the processing of the pore formation in the membranes which could have eventually limited the ability of cell attachment and growth. Data indicate good cell viability in the patterned areas of the membrane regardless of the type of PET membrane modification. 

The membranes here realized and characterized exhibit nearly regular pore position and size combined with customized shape and thickness which could support cell attachment, growth and alignment over the pores. The overall appearance of larger pore defects through which the cells could migrate to the opposite side of the membrane is rare and can be further minimized. Such membrane properties are promising, e.g., for the design of porous septa in microfluidic and organ-on-chips devices, where precise control of the size and density of pores is critical for final applications. Work is in progress to fabricate perfect regular defect-free arrays of pores with tailored sizes and shapes to enable their wider use in microfluidics and biological assays.

## Data Availability

Not applicable.

## References

[1] Kolf W.J., Berk H.T., ter WElle M., van der Ley A.J., van Dijk E.C., van Noordwijk J. (1997). The Artificial Kidney: A Dialyzer with Great Area. 1944. J. Am. Soc. Nephrol..

[2] Baker R.W. (2004). Membrane Technology and Applications.

[3] Fleischer R.L., Price P.B., Symes E.M. (1964). Novel Filter for biological materials. Science.

[4] Kamrani H., Nosrati A. (2018). Fabrication of Nanofiber Filtration Membranes Using Polyethylene Terephthalate (PET): A Review, Journal of Membrane. Sci. Technol..

[5] Yeszhanov A.B., Korolkov I.V., Dosmagambetova S.S., Zdorovets M.V., Güven O. (2021). Recent Progress in the Membrane Distillation and Impact of Track-Etched Membranes. Polymers.

[6] Pasman T., Baptista D., van Riet S., Truckenmüller R.K., Hiemstra P.S. (2021). Development of an In Vitro Airway Epithelial–Endothelial Cell Culture Model on a Flexible Porous Poly(Trimethylene Carbonate) Membrane Based on Calu-3 Airway Epithelial Cells and Lung Microvascular Endothelial Cells. Membranes.

[7] Su N., Hao Y., Wang F., Hou W., Chen H., Luo Y. (2021). Mesenchymal stromal exosome–functionalized scaffolds induce innate and adaptive immunomodulatory responses toward tissue repair. Sci. Adv..

[8] Jiang L., Shu L., Zheng J., Li Y., Huang H. (2019). Recent Progress in Microfluidic Models of the Blood-Brain Barrier. Micromachines.

[9] Chen X., Liu C., Muok L., Zeng C., Li Y. (2021). Dynamic 3D On-Chip BBB Model Design, Development, and Applications in Neurological Diseases. Cells.

[10] Apel P.Y. (1995). Heavy particle tracks in polymers and polymeric track membranes. Radiat. Meas..

[11] Apel P.Y. (2001). Track etching technique in membrane technology. Radiat. Meas..

[12] Kutuzau M., Kozlovskiy A., Borgekov D., Kenzhina I., Zdorovets M.V., Chernik A., Alisienok O., Shumskaya A., Kaniukov E. (2019). Optimization of PET Ion-Track Membranes Parameters. Mater. Today Proc..

[13] Cutroneo M., Havranek V., Torrisi L., Svecova B. (2016). Ion micro beam, promising methods for interdisciplinary research. JINST.

[14] Apel Y., Blonskaya I.V., Ivanova O.M., Kristavchuk O.V., Lizunov N.E., Nechaev A.N., Orelovich O.L., Polezhaeva O.A., Dmitriev S.N. (2020). Creation of Ion-Selective Membranes from Polyethylene Terephthalate Films Irradiated with Heavy Ions: Critical Parameters of the Process. Membr. Membr. Technol..

[15] Hnatowicz V. (2003). Role of scission and cross-linking in latent track formation in polymers. Nucl. Instrum. Methods Phys. Res. B.

[16] Nebogatikova N.A., Antonova I.V., Erohin S.V., Kvashnin D.G., Olejniczak A., Volodin V.A., Skuratov A.V., Krasheninnikov A.V., Sorokin P.B., Chernozatonskii L.A. (2018). Nanostructuring few-layer graphene films with swift heavy ions for electronic application: Tuning of electronic and transport properties. Nanoscale.

[17] Chung H.H., Mireles M., Kwarta B.J., Gaborski T.T. (2018). Use of Porous membranes in tissue barrier and co-culture models. Lab Chip..

[18] Ziel R., Haus A., Tulke A. (2008). Quantification of the pore size distribution (porosity profiles) in microfiltration membranes by SEM, TEM and computer image analysis. J. Membr. Sci..

[19] Mukaibo H., Horne L.P., Park D., Martin C.R. (2009). Controlling the Length of Conical Pores Etched in IonTracked Poly(ethylene terephthalate) Membranes. Small.

[20] Ma Y. (2022). Multiscale Fractal Characterization of Pore Structure for Coal in Different Rank Using Scanning Electron Microscopy and Mercury Intrusion Porosimetry. Processes.

[21] Blonskaya I.V., Kristavchuk O.V., Nechaev A.N., Orelovich O.L., Polezhaeva O.A., Apel P.Y. (2021). Observation of latent ion tracks in semicrystalline polymers by scanning electron microscopy. J. Appl. Polym. Sci..

[22] Cutroneo M., Havranek V., Mackova A., Semian V., Torrisi L., Calcagno L. (2016). Micro-patterns fabrication using focused proton beam lithography. Nucl. Instrum. Methods Phys. Res. B Beam Interact. Mater. At..

[23] Macková A., Malinský P., Cutroneo M., Havránek V., Voseček V., Flaks J., Semián V., Vonka L., Zach V., Bém P. (2021). Small accelerators and their applications in the CANAM research infrastructure at the NPI CAS. Eur. Phys. J. Plus.

[24] Ziegler J.F., Ziegler M.D., Biersack J.P. (2010). SRIM—The stopping and range of ions in matter (2010). Nucl. Instrum. Methods Phys. Res. B Beam Interact. Mater. At..

[25] Jencic J.B., Vavpetic P., Kelemen M., Vencelj M., Vogel-Mikuš K., Kavčič A., Pelicon P. (2019). MeV-SIMS TOF Imaging of organic tissue with continuous primary beam. J. Am. Soc. Mass Spectrom..

[26] https://www.ni.com/cs-cz/support/downloads/software-products/download.labview-vi-analyzer-toolkit.html#411412.

[27] Crawford W.T., DeSorbo W., Humphrey J.S. (1968). Enhancement of track etching rates in charged particle-irradiated plastics by a photo-oxidation eOect. Nature.

[28] Cutroneo M., Havranek V., Torrisi A., Mackova A., Malinsky P., Slepicka P., Sofer Z., Torrisi L. (2020). Polydimethylsiloxane–graphene oxide composite improving performance by ion beam irradiation. Surf. Interface Anal..

[29] Cutroneo M., Havranek V., Semian V., Torrisi A., Mackova A., Malinsky P., Silipigni L., Slepicka P., Fajstavr D., Torrisi L. (2021). Porous polydimethylsiloxane filled with graphene based material for biomedicine. J. Porous Mater..

[30] Hnatowicz V. (2016). On the structure of etched ion track in polymers. Radiat. Phys. Chem..

[31] Vacik J., Cervena J., Hnatowicz V., Fink D., Apel P.Y., Strauss P. (1998). Ion transmission-new technique for analysis of ion tracks in polymers. Nucl. Instrum. Methods Phys. Res. B Beam Interact. Mater. At..

[32] Dutt S., Apel P., Lizuno N., Notthoff C., Wen Q., Trautmann C., Mota-Santiago P., Kirby N., Kluth P. (2021). Shape of nanopores in track-etched polycarbonate membranes. J. Membr. Sci..

[33] Apel P., Bashevoy V., Blonskaya I., Lizunov N., Orelovitch O., Trautman C. (2016). Shedding light on the mechanism of asymmetric track etching: An interplay between latent track structure, etchant diffusion and osmotic flow. PCCP.

[34] Apel P.Y., Blonskaya I.V., Orelovitch O.L., Dmitriev S.N. (2009). Diode-like ion track asymmetric nanopores: Some alternative methods of fabrication. Nucl. Instrum. Methods Phys. Res. B Beam Interact. Mater. At..

